# An International Survey of Patients with Ataxia: Trends in Patient-Reported Symptoms

**DOI:** 10.1007/s12311-026-02012-3

**Published:** 2026-05-02

**Authors:** Celeste Suart, Aimee Alcott, Lori Shogren, Kelsey Trace, Liana S. Rosenthal, Lauren Moore

**Affiliations:** 1 National Ataxia Foundation, Minneapolis, MN USA; 2 Department of Biochemistry and Biomedical Sciences, McMaster University, Hamilton, ON Canada; 3 Department of Neurology, Johns Hopkins School of Medicine, Baltimore, MD USA

**Keywords:** Ataxia, Neurodegenerative disorders, Patient preference, Quality of life, Survey, Signs and symptoms

## Abstract

Ataxia disorders have complex symptomology and few treatment options. Limited information is available on symptoms and quality of life with ataxia from the perspective of patients and caregivers. We aimed to assess whether trends in symptoms and quality of life varied by ataxia type and disease stage. We conducted an anonymous, international survey of 680 National Ataxia Foundation Members, including 587 people with ataxia and 93 caregivers. Data was analyzed for the whole group, by ataxia type, and by Functional Disability Stage (FDS). Respondents self-reported the first symptoms experienced, symptoms currently experienced, and the one symptom that currently has the greatest impact on their life. Impaired balance (51%, *n* = 343) and coordination (24%, *n* = 161) were the most reported first symptoms noticed by respondents across ataxia types. Impaired balance (92%, *n* = 627), impaired coordination (80%, *n* = 546), and fatigue (70%, *n* = 479) were the most prevalent symptoms across ataxia types and FDS. Impaired balance (65%, *n* = 443) was the most reported symptom as having the greatest impact on respondents’ day-to-day life. While there were commonalities between FDS and ataxia types, some subtype differences emerged amongst self-reported first symptom, symptom prevalence, and symptoms impacting daily living. This study highlights trends in symptomology across ataxia types and disease stages from the perspective of people with ataxia and caregivers. While similarities exist across ataxia types, variation in which symptoms most impact quality of life across disease stages underscores the importance of selecting patient-relevant clinical trial outcome measures and endpoints. These findings can further guide clinicians in prioritizing symptom management and treatment planning, while supporting more meaningful, patient-centered discussions about disease impact.

**Supplementary Information:**

The online version contains supplementary material available at 10.1007/s12311-026-02012-3.

## Introduction

Ataxia is the impaired coordination of voluntary movement that can result from multiple underlying etiologies [1, 2]. Patients are often diagnosed based on the characteristic wide-based gait and balance difficulties associated with ataxia [2]. Other common symptoms of cerebellar ataxia include impairments with speech and swallowing, eye movement and vision, cognition, and mood [2, 3]. These symptoms can significantly impact the quality of life experienced by ataxia patients and their families. The diverse underlying causes of ataxia, and the variety of symptomatic presentations, often result in challenges with diagnosis and treatment [3].

Evaluation of ataxia symptoms has largely relied on clinician-evaluated or administered measures, such as MRI to detect changes in cerebellar atrophy [4], digital biomarker sensors [5, 6], neuropsychiatric and cognitive assessments [7, 8], and motor function scales such as the Scale for Assessment and Rating of Ataxia (SARA) [9] or modified Friedreich Ataxia Rating Scale (mFARS) [10]. However, there has been increased use of patient-reported outcome measures (PROMs), such as the PROM-ataxia or FA-ADL [11–14], in both clinical and research settings to better understand and quantify the patient experience of ataxia.

This increased use of PROMs follows a trend of patient-centered healthcare, wherein the lived experiences of patients and family members are valued alongside clinical judgment [15, 16]. Patient-centered conversations shed light on disease burdens, quality of life, and treatment preferences [15–17]. This information can be beneficial to clinicians during the diagnostic process as well as when discussing goals of care and symptom-focused treatment options following an ataxia diagnosis. Moreover, regulatory agencies are placing increasing importance on incorporating patient input into the development and testing of new therapeutics [18].

The National Ataxia Foundation (NAF) was established in 1957 to help people with ataxia and their families. The mission of the NAF is to accelerate the development of treatments and a cure while working to improve the lives of those living with ataxia. With over 20,000 members worldwide, the NAF is one of the largest international ataxia patient advocacy organizations. Due to its size and reach, the NAF is uniquely positioned to gather substantial, multifaceted patient and caregiver input through tools such as surveys. In January 2025, the NAF conducted a robust community survey to maintain programming and service alignment with community needs. While gathering feedback on the support services offered to NAF members via online survey, there was a secondary goal to capture trends in symptomology across ataxia types and disease stages. The objective of this study was to assess whether trends in symptoms and quality of life impacts existed through soliciting responses from the large number of ataxia patients and caregivers in the NAF membership database.

## Patients and Methods

This study was reviewed by the North Star Review Board (Application #NB400246). It was granted an exemption determination from the Common Rule under 45 CFR 46.104(d)(2), due to the anonymous nature of the study.

### Study Population

The membership of the NAF is diverse, including patients, caregivers, healthcare practitioners, researchers, industry professionals, and advocacy workers. Members may register with NAF free of charge. Registration is completed online, with required information including contact information, affiliation with ataxia, such as being a patient or caregiver, ataxia type if applicable, and selecting the types of emails they would like to receive from NAF. Members can select from 83 different ataxia types during registration, including an open text box option for diagnoses not currently represented. The NAF currently has members registered in 139 countries. Members can opt out of receiving emails while remaining a member.

### Survey Design and Administration

Two emails with information about the study and a link to participate were shared with all eligible NAF members, approximately 13,484 individuals, between January 6 to January 20, 2025. NAF members were eligible to participate if they were an ataxia patient or caregiver and had given the NAF permission to contact them. Social media advertisement was used to remind individuals to check their email to access the survey link. A total of 680 individuals completed the survey, representing a response rate of 5%. Informed consent was obtained immediately prior to participants accessing the survey. If someone declined to participate on the informed consent webpage or only partially filled out the required portion of the survey, this was treated as withdrawal from the study.

This anonymous survey was designed to provide descriptive, observational, cross-sectional information about the NAF members. Sections of the survey included demographic information, ataxia diagnosis and symptoms, social determinants of health information, and feedback on various NAF services. The data presented herein focuses on sections related to symptomology from the perspective of patients. Caregiver respondents were asked to respond to these questions on behalf of their loved one with ataxia. The survey was only available in English.

We also incorporated validated clinical assessments, specifically the Functional Disability Stage (FDS) and the Perception of Symptom Burden Scale. Participants were asked to self-report their FDS. This score describes mobility and independence and was initially developed for Friedreich Ataxia clinical research [19]. However, its use has expanded to other forms of ataxia [20, 21]. The FDS is a six-point scale, ranging from zero (no symptoms) to six (full-time use of a wheelchair or remains in bed, dependent on others for all activities of daily living).

We used a modified version of the Perception of Symptom Burden scale previously used within a Friedreich Ataxia study cohort to examine trends in symptomology across ataxia types [22]. The Perception of Symptom Burden scale is a multiple-choice list of seven common ataxia symptoms described in terms familiar to patients, such as ‘impaired coordination’ instead of ‘dysmetria [22]. This scale was initially designed as a forced-choice scale to determine what percentage of Friedreich Ataxia patients and caregivers would select a particular symptom as most burdensome. Modifications included removing the response option of scoliosis, due to the rarity of this symptom outside of Friedreich Ataxia, and the separation of speech, vision, and hearing impairment symptoms due to known variations in these symptoms by ataxia type [3, 23]. This scale was selected due to informal feedback from NAF members on its ease of use. The Perception of Symptom Burden was used to gauge respondents’ first symptom, currently experienced symptoms, and the symptom of greatest burden on daily life. Respondents could select a single response on the Perception of Symptom Burden for their first symptom and the symptom of greatest burden [22]. Respondents could select multiple options for the symptoms they are currently experiencing.

### Statistical Analysis

Data cleaning and descriptive statistics were completed using Microsoft Excel. Normality testing and comparative analysis were completed using GraphPad Prism (Version 10.6.1). Continuous numerical data was tested for normality using the D’Agostino-Pearson normality test, followed by comparative analysis with the Mann–Whitney U test due to the non-normal nature of these data. Nominal data were analyzed via Chi-squared test and Fisher’s exact test as indicated, depending on sample size. Bonferroni correction for multiple comparisons was completed using R (Version 4.5.3). For all tests, the P value was considered significant at < 0.05.

Comparative analysis was conducted for subgroups that represented 4% or more of the total sample population. This cut-off was decided amongst the research team, prior to the launch of the survey, to show the variety of ataxia types reflected in this study while also keeping the number of subgroups manageable. Analysis groups were defined using three characteristics: ataxia category, specific ataxia subtype, and FDS (SI Fig. 1). Four ataxia categories were used for analysis groups, including dominant genetic ataxia, recessive or X-linked genetic ataxia, acquired ataxia from injury or illness, and sporadic ataxia, also known as idiopathic ataxia, which includes diagnoses of ataxia given to individuals after known genetic and acquired causes have been ruled out. Respondents in each ataxia category included all those with ataxia subtypes that fall into that category, regardless of whether a subsequent subtype-specific analysis was completed. When comparing a specific subtype to an ataxia category, respondents of that subtype were removed for statistical analysis. Eight ataxia subtypes were used for analysis groups, including spinocerebellar ataxia (SCA) type 6, SCA3, SCA27B, SCA8, SCA2, RFC1 Ataxia, Friedreich Ataxia (FA), and ataxia of unknown origin without family history. While there are likely multiple underlying etiologies that are represented within the ataxia of unknown origin without family history grouping, we were interested in examining whether any similarities in symptomology existed within. Respondents who were unsure of what category their ataxia type belonged to were included in total and FDS disease stage analysis, with no ataxia type group analysis completed. For subgroup analysis focusing on disease stage using FDS measures, Stages 0 and 1, as well as Stages 5 and 6, were grouped together due to limited respondent numbers in Stages 0, 1, and 6. Concordance testing between Ataxian and caregiver responses was used to assess whether caregivers would have an accurate assessment of their loved one’s symptoms, or if there were significant differences in symptomology trends reported by Ataxians and caregivers.

## Results

Respondents self-reported the ataxia diagnosis of themselves or their loved one using a branching logic screen (Table 1). A total of 68 distinct diagnoses were reported. The study sample comprised 369 respondents with a dominant genetic ataxia (such as Spinocerebellar Ataxia, Episodic Ataxia), 111 respondents with a recessive genetic ataxia (such as RFC1 Ataxia, Friedreich Ataxia, Spastic Paraplegia 7, Ataxia with Oculomotor Apraxia), 29 respondents with an acquired ataxia (such as Gluten Ataxia, Paraneoplastic Ataxia, Post-Stroke Ataxia) and 94 respondents with a sporadic ataxia (Ataxia with unknown origin, SCA type Unknown) (Table 1). Of note, 11.3% of respondents, while confident they or their loved one had an ataxia diagnosis, were unsure what type of ataxia they had or how it was classified. People with ataxia, known as Ataxians, and caregivers were equally likely to be unsure of their ataxia diagnosis category (*P* = 0.8682, Fisher’s Exact Test). The majority of respondents, or their loved ones, reported a Functional Disability Stage between 2 and 4, representing symptoms ranging from mild to moderate disability (Table 1). Caregiver respondents were significantly more likely to be responding on behalf of a loved one in stage 5 or 6, representing severe disability (*P* = 0.0012, Fisher’s Exact Test) (Table 1).

**Table 1:** Medical Characteristics of Ataxians. Caregiver respondents are included in table according to the indicated Functional Disease Stage (FDS) and ataxia type of the individual cared for. Mean value and standard deviation in years are provided for age at onset of symptoms and disease duration. *N* = 680

Functional Disability Stage (FDS)	%	*N*	Age at Onset of Symptoms (Years)	Disease Duration (Years)
Stage 0	0.4%	3	42.7 ± 12.5	0
Stage 1	4.0%	27	41.2 ± 19.2	7.7 ± 11.5
Stage 2	22.1%	150	42.7 ± 20.8	11.6 ± 11.4
Stage 3	25.9%	176	43.6 ± 19.6	17.6 ± 14.6
Stage 4	31.2%	212	43.8 ± 17.5	19.3 ± 14.2
Stage 5	14.1%	96	39.4 ± 18.6	21.9 ± 15.9
Stage 6	2.4%	16	47.1 ± 21.0	15.9 ± 13.9
Ataxia Type				
Genetic Dominant	54.3%	369	44.3 ± 17.6	15.4 ± 13.3
SCA6	13.4%	91	53.4 ± 11.7	12.8 ± 11.0
SCA3	10.4%	71	43.3 ± 12.1	11.8 ± 8.0
SCA27B	6.5%	44	60.6 ± 11.4	11.2 ± 9.0
SCA8	4.4%	30	32.4 ± 19.6	20.6 ± 14.6
SCA2	4.1%	28	40.9 ± 14.5	19.2 ± 17.5
Genetic Recessive	16.3%	111	34.5 ± 20.3	20.2 ± 15.0
RFC1 Ataxia	4.9%	33	48.5 ± 14.8	18.4 ± 12.8
Friedreich Ataxia	3.5%	24	27.5 ± 19.6	16.3 ± 9.7
Acquired	4.3%	29	46.0 ± 19.4	16.0 ± 16.4
Sporadic	13.8%	94	44.1 ± 20.5	20.5 ± 15.4
Ataxia of Unknown Origin, without Family History	5.0%	34	38.5 ± 18.1	18.1 ± 15.7
Unsure of Type	11.3%	77	45.6 ± 19.7	19.1 ± 15.8

On average, it took Ataxians (27.2 min), significantly longer to complete the survey than caregivers (20.4 min) (*P* < 0.0001, Mann-Whitney Test). No significant differences in completion times were detected between ataxia subtypes. The majority of survey respondents were female, married, unemployed, and living in the United States in suburban environments (Table 2). These characteristics are comparable with known demographic trends within NAF membership regarding location. Most often, caregivers reported caring for their spouse (42.6%), child (29.8%), or parent (16.0%), with 84.9% of respondents caring for someone over the age of 18. Respondents with ataxia were significantly more likely to be unemployed compared to caregiver respondents (*P* = 0.0003, Fisher’s Exact Test). Caregiver respondents answered questions on disease stage and symptomology from the perspective of their loved one with ataxiafor whom they provide care.

**Table 2:** Sociodemographic characteristics of respondents. *N* = 680

Sociodemographic Variable	%	*N*
Age	61.0 ± 13.9 (18—92)	
Gender		
Female	63.4%	431
Male	36.0%	245
Non-Binary	0.4%	3
Prefer not to Answer	0.1%	1
Respondent Type		
Ataxian	86.3%	587
Caregiver	13.7%	93
Ataxian Relationship to Caregiver		
Spouse	42.6%	40
Child	29.8%	28
Parent	16.0%	15
Sibling	7.4%	7
Extended Family	2.1%	2
Other	1.1%	1
Marital Status		
Married	65.6%	446
Single	22.5%	153
Widowed	6.2%	42
Living with a Partner	4.6%	31
Prefer not to Answer	1.2%	8
Employment Status		
Not employed	66.5%	452
Employed full-time	17.6%	120
Employed part-time	6.9%	47
A homemaker	4.9%	33
A full-time student	0.9%	6
Prefer not to Answer	3.2%	8
Country		
USA	80.7%	549
Canada	6.6%	45
United Kingdom	2.9%	20
Australia	2.4%	16
Ireland	1.0%	7
New Zealand	0.9%	6
Other	5.4%	37
Urbanicity		
Suburban	52.2%	355
Urban	26.3%	179
Rural	20.4%	139
Prefer not to Answer	1.0%	7

### Impaired Balance Most Reported First Symptom Experienced by Ataxians

**Fig. 1:**
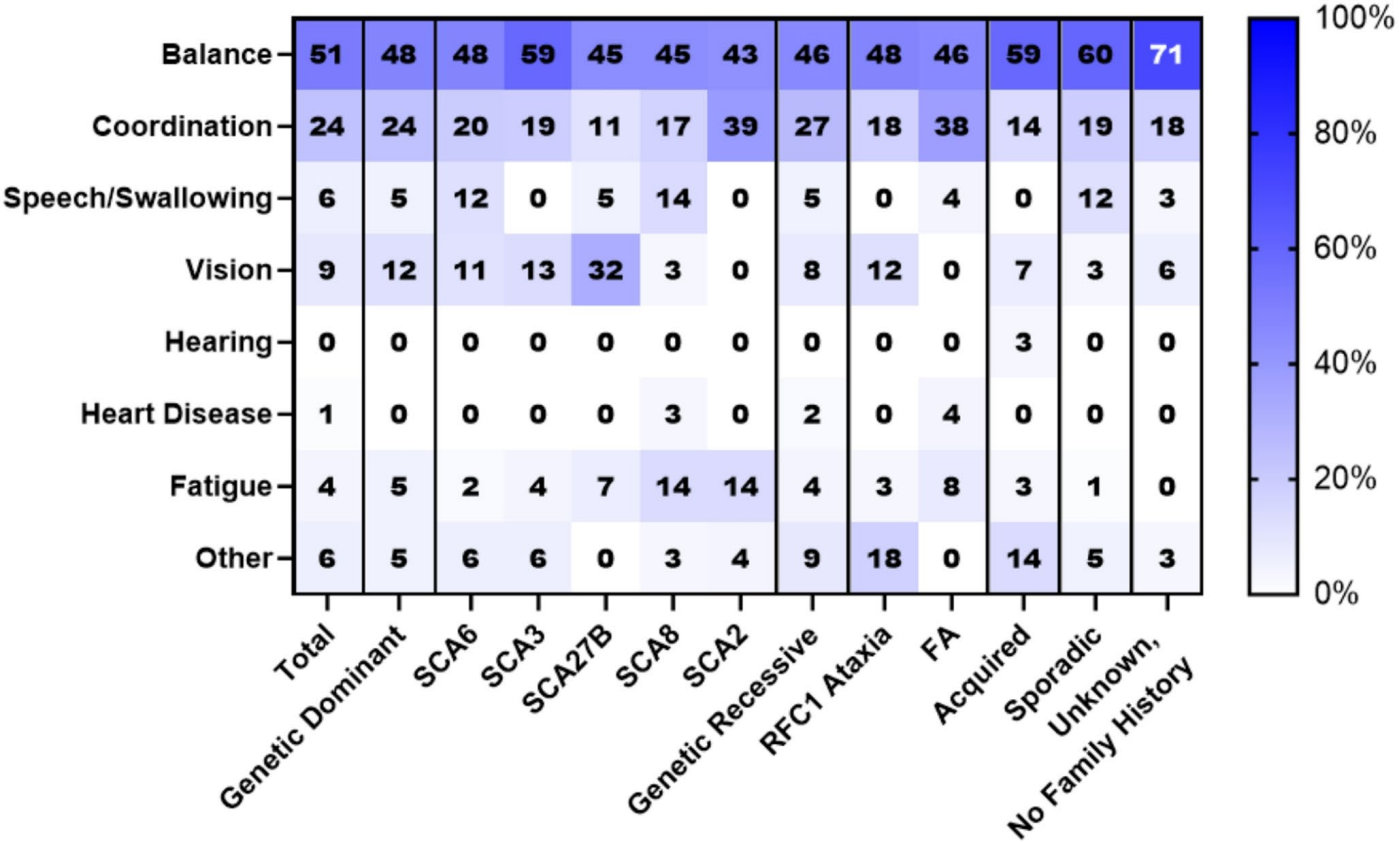
First symptom self-reported, stratified by ataxia type. Value in each cell indicates the percentage of respondents reporting this symptom

Respondents markedly reported balance impairment, including challenges with walking, as the first symptom they or their loved one first remembered experiencing (51%, *n* = 343, Fig. 1). For the total sample, as well as most ataxia categories and subtypes, impaired coordination was the second most-reported first symptom (11–38%, *n* = 5-161). This includes both fine motor and gross motor challenges. Notably, there were no significant differences in first symptoms reported between Ataxians and caregivers (*P* = 0.99, Chi-Squared Test).

However, one subtype-specific difference in the first symptom experienced was reported. Respondents with SCA27B were significantly more likely than all other respondents (*P* = 0.0007, Fisher’s Exact Test) and respondents with other forms of dominant genetic ataxia (*P* = 0.0014, Fisher’s Exact Test) to report impaired vision as their first symptom.

### Impaired Balance, Impaired Coordination, and Fatigue are Prevalent Ataxia Symptoms

**Fig. 2:**
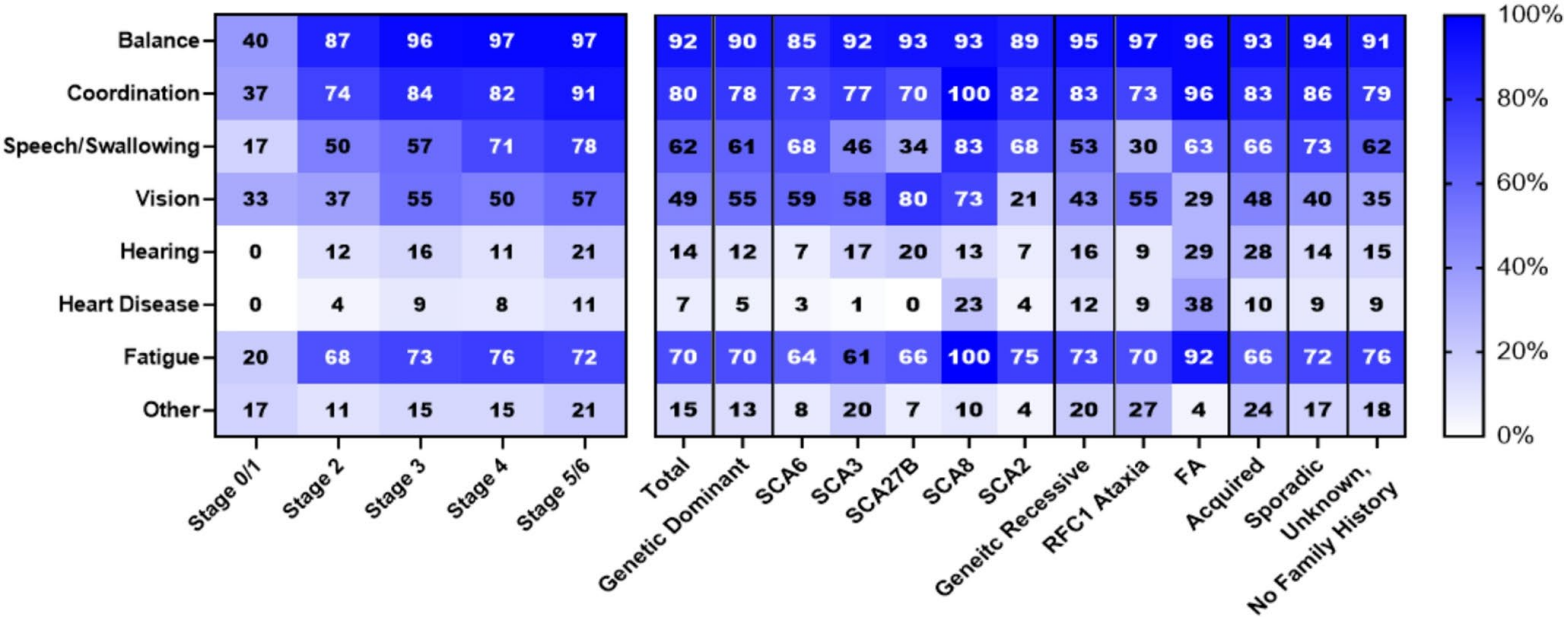
Self-reported symptom prevalence, stratified by functional disease stage and ataxia type. Value in each cell indicates the percentage of respondents reporting this symptom

Next, we asked participants about symptoms they or their loved ones were currently experiencing. Participants could select as many symptoms as they wanted. Overall, impaired balance (92%, *n* = 627), impaired coordination (80%, *n* = 546), and fatigue (70%, *n* = 479) were the most reported symptoms (Fig. 2). Impaired speech or swallowing (62%, *n* = 419) and impaired vision (49%, *n* = 332) were also prevalent symptoms within the sample population, with impaired hearing (14%, *n* = 93), other symptoms (15%, *n* = 103), or heart disease (7%, *n* = 49) being less frequently reported. Examples of other symptoms include tremor, neuropathy, cough, cognitive changes, spasticity, impaired sleep, and autonomic symptoms. There were no significant differences in symptoms reported between Ataxians and caregivers (*P* = 0.99, Chi-Squared Test). Additionally, there were no significant differences in symptom prevalence reported between Functional Disability Stages (*P* = 0.99, Chi-Squared Test).

There were no significant differences in the composition of symptom prevalence between dominant genetic ataxia, recessive genetic ataxia, acquired ataxia, and sporadic ataxia (*P* = 0.99, Chi-Squared Test). However, certain subtypes of ataxia had significant differences in the composition of reported symptom prevalence. This analysis compares all symptoms reported by respondents simultaneously. The composition of reported symptom prevalence for FA was significantly different than those of other recessive genetic ataxia respondents (*P* = 0.006, Chi-Squared Test). The composition of reported symptom prevalence for SCA27B (*P* = 0.044, Chi-Squared Test) was significantly different than those of other dominant genetic ataxia respondents.

### Impaired Balance Has a Meaningful Impact on Day-To-Day Life

**Fig. 3:**
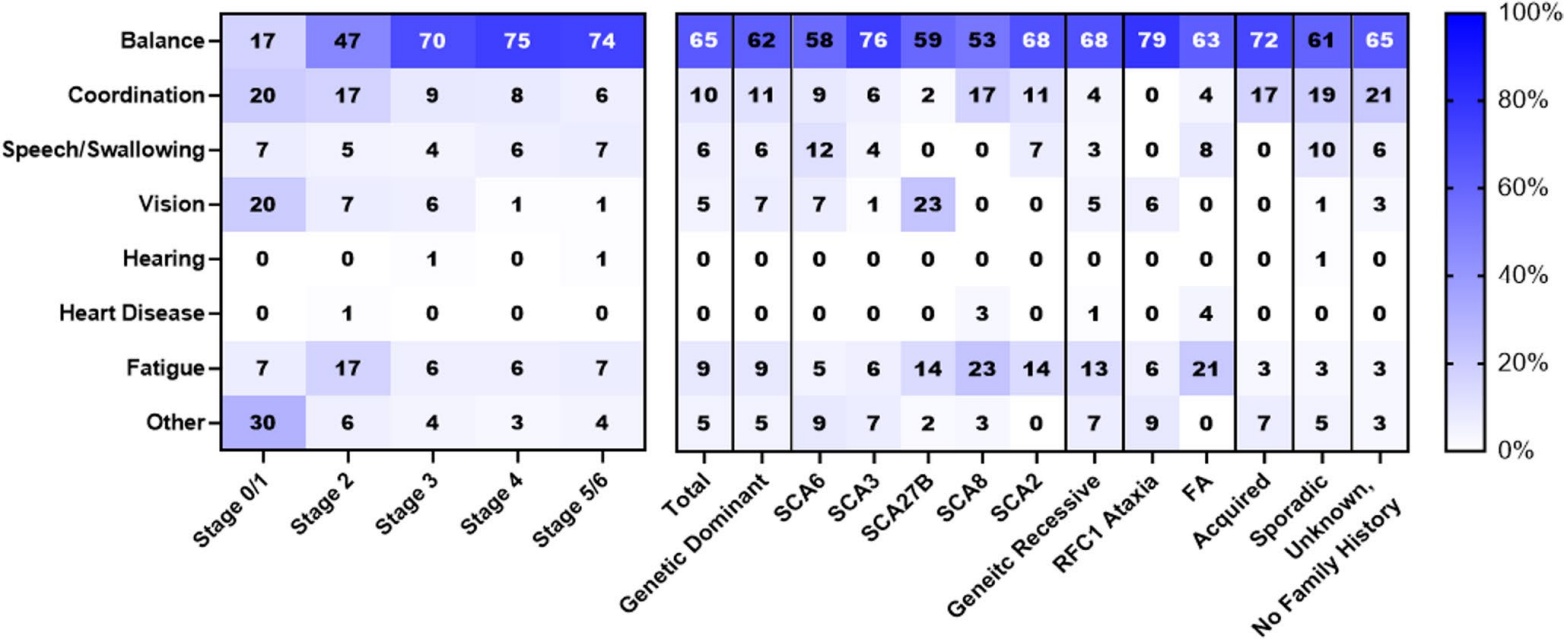
Symptom with the greatest impact on day-to-day life, stratified by functional disease stage and ataxia type. Value in each cell indicates the percentage of respondents reporting this symptom

Finally, we asked participants which ataxia symptom has the greatest impact on their or their loved one’s day-to-day life. Impaired balance was predominantly the most selected symptom for the greatest impact across ataxia categories and ataxia subtypes (Fig. 3). There were no significant differences in symptoms selected by Ataxians and caregivers (*P* = 0.99, Chi-Squared Test).

However, there was a significant relationship between the reported symptom of greatest impact and disease stage (*P* = 0.0007, Chi-Squared Test).In stage 0 and stage 1, balance only accounted for 17% (*n* = 5) of respondents’ symptom of greatest impact, while other symptoms, including tremor, dystonia, dizziness, and spasticity, accounted for 30% (*n* = 30) of responses. Only 47% (*n* = 71) of stage 2 respondents indicated that impaired balance had the greatest impact on their day-to-day life, followed by fatigue (17.3%, *n* = 26) and impaired coordination (16.7%, *n* = 25). There were no significant differences in responses from respondents in Stage 3, Stage 4, Stage 5, and Stage 6, with impaired balance as the primary symptom of greatest impact (*P* = 0.99, Chi-Squared Test).

There was a significant relationship between the reported symptom of greatest impact and ataxia category, largely driven by differences between sporadic ataxia respondents and those from other categories (*P* = 0.0378, Chi-Squared Test). Respondents with SCA27B also reported significantly different composition of symptoms of greatest impact, particularly impaired vision and fatigue, when compared to other dominant types of genetic ataxia (*P* = 0.0444, Fisher’s Exact Test).

## Discussion

This study found that trends exist across ataxia types for the first symptom experienced, prevalence of symptoms, and symptoms that have the greatest impact on day-to-day life. While there were commonalities across disease stages and ataxia types, some subtype differences emerged. Our findings are consistent with medical literature characterizing the variety of ataxia types covered in the survey [2, 3, 23–25]. Further, they echo findings from other ataxia patient advocacy organizations [24]. These reports of symptoms were generally consistent between Ataxians and caregivers, which is evidence to suggest that caregiver respondents were accurately reporting the experiences of their loved ones in this study.

Interestingly, 11.3% of respondents, even with explanatory text included in the survey, were unsure what type of ataxia diagnosis they or their loved one had or how a diagnosis fit into categories such as genetic, acquired, or sporadic ataxia. This number is likely an underestimate, as the NAF received several emails and phone calls from community members asking for clarification and support to answer this survey question. This is an important consideration for researchers advertising studies to the ataxia community, as inclusion or exclusion criteria based on diagnosis terminology may not be clear or accessible to some patients and caregivers. Additionally, it is important to consider the role of clinicians in clearly communicating ataxia diagnosis categories and subtypes by defining the diagnosis in both technical and patient-centered terms, explaining how it fits within broader groupings, and outlining what it means for symptoms, progression, and care. We must also consider how the redistribution of these individuals to a specific ataxia diagnosis or grouping may have shifted our reported results.

Unsurprisingly, impaired balance was the most reported first symptom that patients remember experiencing. While impaired coordination was the second most-reported first symptom for most ataxia categories and subtypes, some important differences were identified. Impaired vision was the second most reported symptom by respondents with SCA27B, mirroring clinical findings around the prevalence of downbeat nystagmus and other cerebellar-oculo-motor signs [26]. Variances in the first symptom differences between ataxia subtypes show how subtle differences in early presentation can help differentiate between ataxia etiologies during the diagnostic process.

There was a surprising amount of homogeneity amongst the gathered symptom prevalence data. With the exception of FA and SCA27B, there was limited significant variation in self-reported symptoms between disease stage, ataxia category, or ataxia subtype across the types. This highlights the potential impact of symptom-focused interventions for ataxia, such as exercise interventions improving balance, coordination, and fatigue [27–30]. Fatigue intervention is of particular interest, as it was a highly reported symptom across subgroups analyzed, known to have a significant impact on quality of life and worsening other symptoms, with few reliable therapeutic interventions [11, 31, 32]. However, one must also consider the difference between a symptom prevalence and symptom burden on a person’s day-to-day life.

Regarding the greatest impact on respondents’ or their loved ones’ day-to-day life, impaired balance continued to be the most reported symptom. There were some reported variations by disease stage. In early disease stages, other symptoms such as impaired coordination, impaired vision, impaired speech, or fatigue had a large impact on the respondent’s day-to-day life. However, as ataxia progressed, and presumably symptoms intensified, impaired balance emerged as the symptom of greatest impact for the majority of respondents. This aligns with previous patient symptom burden and quality of life studies which emphasize the significant therapeutic need for this patient population to participate in quality balance improvement interventions [17, 24]. Nonetheless, it is important to note differences in reported symptoms of greatest impact, such as those reported by patients with SCA27B connected to impaired vision and fatigue as well as patients with sporadic ataxia connected to impaired coordination and impaired speech or swallowing. Considering similarities across ataxia types, as well as variation in which symptoms most impact quality of life across disease stages, may be beneficial when selecting patient-relevant clinical trial outcome measures and endpoints. Attention to these similarities and variations may also assist healthcare practitioners with treatment planning.

There are several limitations to this study. First, this study relies on retrospective self-reported data, which may be impacted by social desirability bias and recall bias [33]. We attempted to minimize the impact of social desirability bias by having the survey anonymous, with options to skip demographic questions to have respondents feel secure in giving their answers. Minimizing the impact of recall bias is more difficult given the retrospective nature of the survey; however, we would argue the results concerning first remembered symptoms still hold value as they would be the same information presented during intake in the clinic.

Second, this survey is by no means comprehensive in terms of symptom measurements included and the population studied. While this study, to our knowledge, is the current largest survey of Ataxians and caregivers in the literature, our sample size only accounts for 5% of eligible participants, and a smaller percentage of the overall population of people with ataxia worldwide. While this response rate is comparable to other large, patient advocacy organization-led surveys [24], it must be considered when making generalizations. There are several potential reasons individuals may have had difficulty completing an online survey, such as limited access to technology or the internet, as well as advanced disease stages. We attempted to mitigate these barriers by using a survey platform which is supported by multiple types of electronic devices, which met WCAG Level AA standards for digital accessibility, and allowed caregivers to respond on behalf of patients who could not complete the survey for whatever reason. However, differences between potential respondents and non-respondents must be considered when generalizing these findings. Given that the results of this study align closely with other survey-based research, case reports, and clinical data on ataxia symptomology, this supports that our findings are likely to have some degree of generalizability.

Third, while the FDS was initially designed as a clinician-rated scale, we employed it as a patient self-reported scale. While self-reported FDS has been used by patient advocacy organizations on an ad hoc basis, no validation studies comparing the concordance of self-reported and clinician-rated FDS have been conducted. Further research into self-reported disease staging tools within ataxia is warranted. Fourth, this survey was only available in English, which would be a barrier for non-English-speaking patients and caregivers. While an English-only survey was employed due to financial constraints and NAF services being provided primarily in English, future studies should seek to provide translation options to increase access for potential participants.

This work builds off previous patient-focused work to better understand the symptom burden of Ataxians [22, 24, 34]. We have extended this work by increasing the sample size of respondents, including more refined data collection tools, incorporating the perspectives of caregivers, and conducting analyses factoring in disease stage and subtype. However, future research in this area has the potential to expand knowledge by incorporating the perspectives of ataxia healthcare professionals, as well as gathering longitudinal data on symptom burden using existing validated patient-reported outcome measures such as the PROM-ataxia or FA-ADL [11–14]. There is also potential for developing validated ataxia type-specific quality of life measures, such as the FA-ADL, or examining how answers may differ between individuals with varying types of ataxia. Other questions, such as the effectiveness of interventions and the timing of interventions, may also be explored through the incorporation of patient feedback.

## Conclusion

This study highlights the similarities and differences in symptomology across ataxia types and disease stages. Key trends in self-reported first symptom, overall symptom prevalence, and symptoms having the greatest impact on day-to-day life were identified in a large cohort of respondents with varying ataxia etiologies. These findings may be valuable for clinicians during the diagnostic process to differentiate between ataxia types and to inform subsequent plan of care, as well as for Ataxians themselves to know they are not alone in their experiences. These data may also be helpful for those designing therapeutic interventions, in both determining what symptomatic changes would have an impact on the lives of Ataxians and selecting patient-relevant outcome measures for clinical trials.

## Electronic Supplementary Material

Below is the link to the electronic supplementary material.

Supplementary Material 1 (DOCX 340 KB)

## Data Availability

The data that support the findings of this study are available from the corresponding author upon reasonable request. The data are not publicly available due to participant privacy and ethical restrictions.
