# Supplementary material for: An International Survey of Patients with Ataxia: Trends in Patient-Reported Symptoms
Source: Cerebellum. 2026 May 2;25(3):69. doi: 10.1007/s12311-026-02012-3 (PMC13135561; doi:10.1007/s12311-026-02012-3)

**Supplementary Information Fig. 1.** Overview of subgroup analysis strategy. Data from this survey was analyzed using overlapping groupings: Ataxia Categories (each including multiple types of ataxia), individual Ataxia Types, and Functional Disability Stage (FDS).


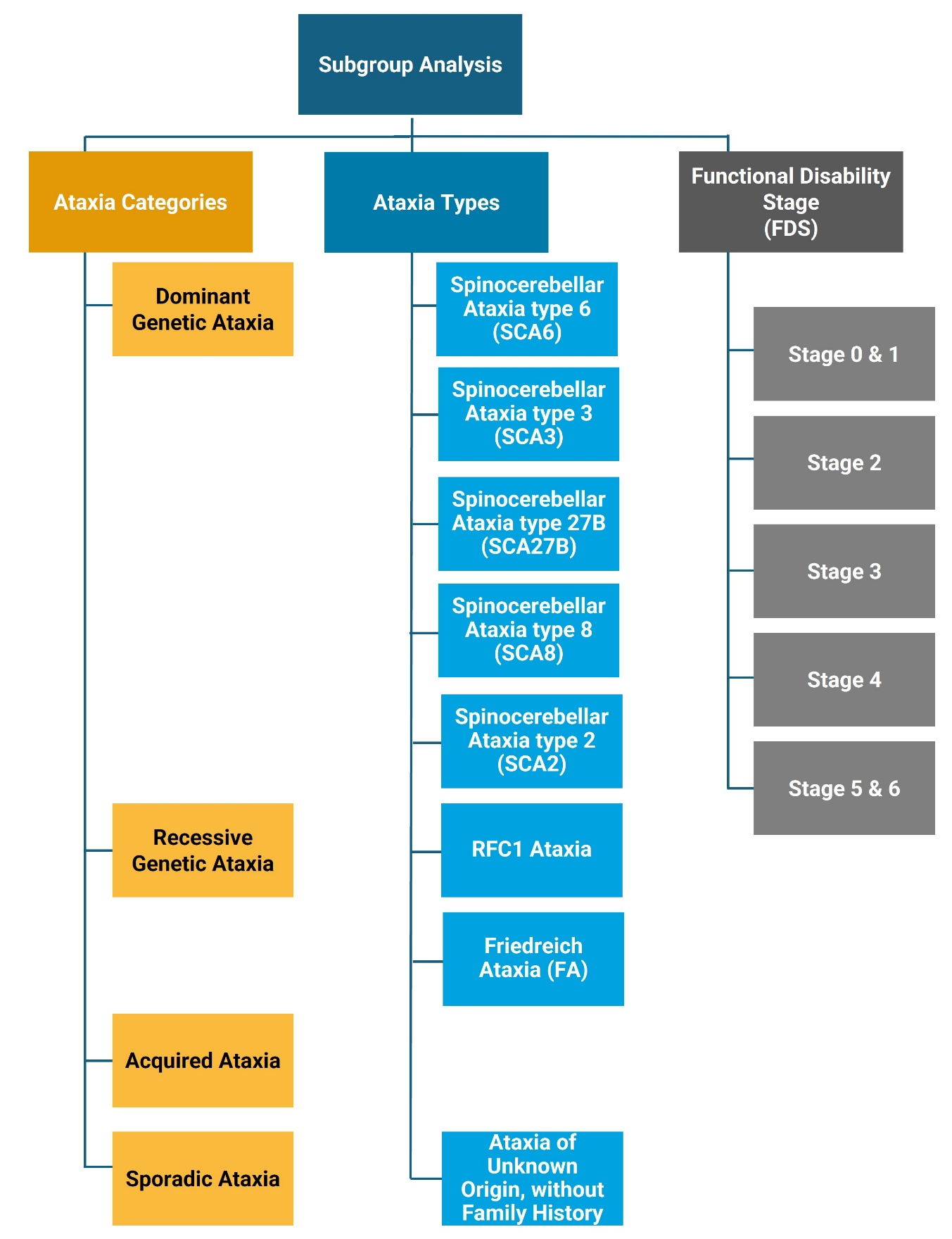

Supplement: Supplementary file 1 — Supplementary Material 1 (DOCX 340 KB) [file 12311_2026_2012_MOESM1_ESM.docx]
